# Reward Based Motor Adaptation Mediated by Basal Ganglia

**DOI:** 10.3389/fncom.2017.00019

**Published:** 2017-03-31

**Authors:** Taegyo Kim, Khaldoun C. Hamade, Dmitry Todorov, William H. Barnett, Robert A. Capps, Elizaveta M. Latash, Sergey N. Markin, Ilya A. Rybak, Yaroslav I. Molkov

**Affiliations:** ^1^Department of Neurobiology and Anatomy, Drexel University College of MedicinePhiladelphia, PA, USA; ^2^Department of Mathematics and Statistics, Georgia State UniversityAtlanta, GA, USA; ^3^Department of Mathematics and Mechanics, Saint Petersburg State UniversitySaint Petersburg, Russia

**Keywords:** basal ganglia, reinforcement learning, dopaminergic reward, motor adaptation, reaching movement

## Abstract

It is widely accepted that the basal ganglia (BG) play a key role in action selection and reinforcement learning. However, despite considerable number of studies, the BG architecture and function are not completely understood. Action selection and reinforcement learning are facilitated by the activity of dopaminergic neurons, which encode reward prediction errors when reward outcomes are higher or lower than expected. The BG are thought to select proper motor responses by gating appropriate actions, and suppressing inappropriate ones. The direct striato-nigral (GO) and the indirect striato-pallidal (NOGO) pathways have been suggested to provide the functions of BG in the two-pathway concept. Previous models confirmed the idea that these two pathways can mediate the behavioral choice, but only for a relatively small number of potential behaviors. Recent studies have provided new evidence of BG involvement in motor adaptation tasks, in which adaptation occurs in a non-error-based manner. In such tasks, there is a continuum of possible actions, each represented by a complex neuronal activity pattern. We extended the classical concept of the two-pathway BG by creating a model of BG interacting with a movement execution system, which allows for an arbitrary number of possible actions. The model includes sensory and premotor cortices, BG, a spinal cord network, and a virtual mechanical arm performing 2D reaching movements. The arm is composed of 2 joints (shoulder and elbow) controlled by 6 muscles (4 mono-articular and 2 bi-articular). The spinal cord network contains motoneurons, controlling the muscles, and sensory interneurons that receive afferent feedback and mediate basic reflexes. Given a specific goal-oriented motor task, the BG network through reinforcement learning constructs a behavior from an arbitrary number of basic actions represented by cortical activity patterns. Our study confirms that, with slight modifications, the classical two-pathway BG concept is consistent with results of previous studies, including non-error based motor adaptation experiments, pharmacological manipulations with BG nuclei, and functional deficits observed in BG-related motor disorders.

## Introduction

The basal ganglia (BG), located in the deep forebrain of most vertebrates, have a complex structure with multiple connected sub-nuclei: the striatum, the globus pallidus (GP), the subthalamic nucleus (STN), and the substantia nigra. The striatum is the primary input structure of the BG and receives many projections from various regions in the cortex. By relaying inputs from the cortex, the BG act as a shared processing unit involved in a broad variety of motor and cognitive behaviors. The GABAergic internal GP (GPi) and substantia nigra pars reticulata (SNr) serve as the output nuclei of BG which send inhibitory projections to the thalamus and to other various brain regions (Hikosaka et al., 1993; Weyand and Gafka, 1998; Takakusaki et al., 2003, 2004; Liu and Basso, 2008). The thalamus has excitatory connections with the cortex thus creating parallel cortico-striatal-thalamo-cortical loops, a network considered critically important for volitional controls (Alexander and Crutcher, 1990; DeLong, 1990; Turner and Anderson, 1997).

The BG are thought to play a pivotal role in establishing appropriate behavioral responses to stimuli by means of reinforcement learning (see Graybiel, 2008 for review). A classical model of the BG assumes that behavioral choices are made by selective release of thalamocortical relay neurons from BG inhibition (Gurney et al., 2001a,b; Frank, 2005; Mannella and Baldassarre, 2015). The output of the BG network depends on synaptic projections from the cortex to medium spiny neurons (MSN) in the striatum. These projections are plastic; their efficacy changes depending on dopaminergic inputs from Substantia Nigra pars compacta (SNc), which code the reward prediction error and underlie positive or negative reinforcement of the action (Frank, 2005).

GABAergic MSNs have two primary phenotypes: D1-type MSNs (D1 MSNs) expressing D1-type dopamine receptors and D2-type MSNs (D2 MSNs) expressing D2-type dopamine receptors (Albin et al., 1989; DeLong, 1990; Kreitzer and Malenka, 2008). Accordingly, increased firing of the SNc dopaminergic neurons leads to long term potentiation (LTP) of excitatory cortical projections to D1 MSNs, and to long term depression (LTD) of the projections to D2 MSNs. D1 MSNs directly project to the GPi (one of BG output stages) and D2 MSNs project to the external GP (GPe). The GABAergic GPe negatively affects the GPi activity by direct inhibitory projections and/or through glutamatergic neurons of STN which receive inhibition from the GPe and send excitatory projections to the GPi (see Nelson and Kreitzer, 2014 for review).

For a behavior to be selected, corresponding thalamic neurons should be released from GPi inhibition, by GPi suppression. This is achieved either by inhibition from corresponding D1 MSNs, or by inhibition from corresponding GPe neurons which, in turn, are released from D2 MSN inhibition. In contrast, to prevent an action from being selected, corresponding GPi neurons should be disinhibited, i.e., neither associated D1 MSN nor STN neurons should be active. Thus, D1 MSNs activation assumes selection of the associated action (GO pathway), while activation of D2 MSNs prevents the associated behavior from execution (NOGO pathway). The GO pathway from the striatum to the GPi is often referred to as “direct,” as D1-MSNs monosynaptically project to the GPi neurons, while the NOGO pathway is “indirect” because D2 MSNs send signals to the GPi via the GPe and STN.

As mentioned, reinforcement of behaviors is mediated by LTP or LTD of cortical projections to D1 and D2 MSNs depending on SNc input. Dopamine release codes the reward prediction error (RPE), which is the difference between the obtained reward and the predicted reward (Hollerman and Schultz, 1998; Schultz, 1998). If the outcome of a behavior is better than expected, the firing rate of SNc neurons increases, and so cortical projections to D1 MSNs are potentiated, while the projections to D2 MSNs get depressed (Centonze et al., 2001). On subsequent trials, MSNs in the GO and NOGO pathways activate to greater and lesser extents, respectively, thus making it more probable for the same action to be selected again. In contrast, if the outcome is worse than expected, D1 projections are depressed, while D2 projections are potentiated, thus decreasing the probability of selection of the performed on subsequent trials.

The classical model of BG was formally implemented in many studies and was successful in explaining various observations in behavioral experiments involving decision-making and reinforcement learning (Gurney et al., 2001a,b; Frank, 2005, 2006; Mannella and Baldassarre, 2015; Wei et al., 2015). The experimental settings used and modeled in such studies include a limited number of possible behavioral outcomes/actions. However, even the simplest behaviors, e.g., pressing a button or following a branch of a maze, are actuated by a very complex musculoskeletal apparatus, involving well-timed activation of multiple muscles.

Most existing BG models include abstract representation of possible actions, while omitting explicit translation of the action choice into an action-specific muscle activation pattern. This translation is important, because any abstract action can be performed in many ways from the motor control standpoint. While the involvement of the BG in abstract action selection is widely accepted, it is still unclear whether the BG directly influence muscle activation patterns, regarding the current environmental constraints. Does the brain prepare a specific context for the BG learning system, and then post-process its output to create a dynamic motor response? Alternatively, are the BG directly involved in the motor pattern formation at a lower level, synthesizing response behavior? The experimental evidence for the latter is the involvement of BG in motor adaptation (Izawa and Shadmehr, 2011; Galea et al., 2015).

To address these questions, we developed a model, which couples the classical BG model with a neuromuscular system controlling planar movements of the arm. The distinguishing feature of the model is the virtually unlimited number of elementary low-level actions, which can be dynamically combined into more abstract behaviors solely based on reinforcement mechanisms. The model is used to simulate abstract action selection experiments (e.g., pressing buttons), as well as reinforcement based motor adaptation tasks. Based on our simulations, we suggest minor modifications to the classical BG concept to resolve the existing controversies concerned with pharmacological manipulations with the BG and effects of certain motor disorders.

## Materials and methods

### Model structure

**Figure 1:**
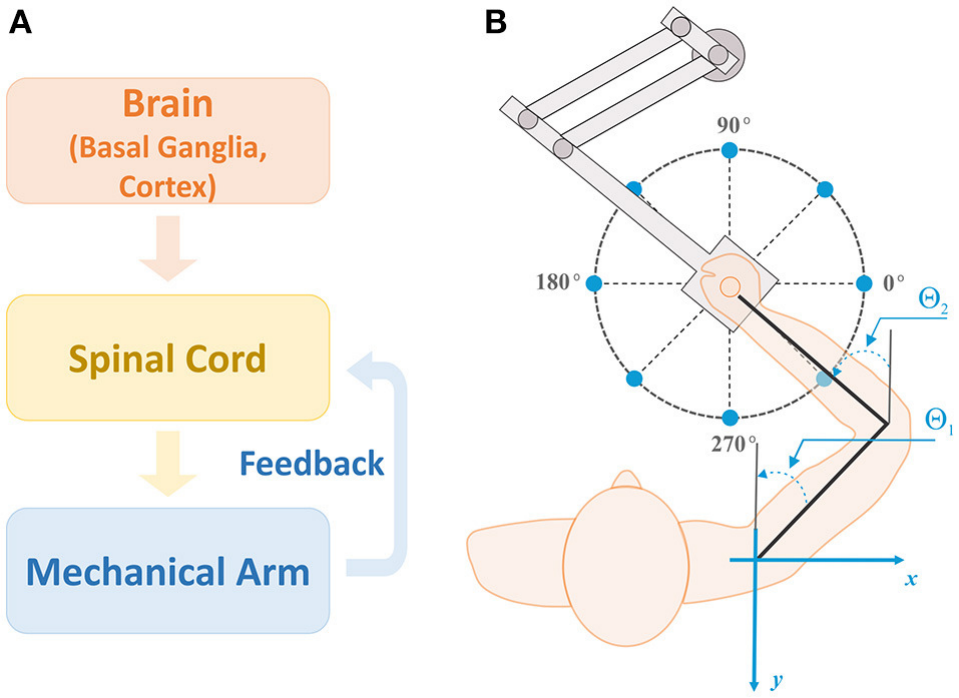
**Model structure and setup for reaching. (A)** Model structure. There are three main modules in the model: brain, spinal cord, and biomechanical arm modules. The brain module consists of the basal ganglia (BG) and cortex, and sends out motor commands into the spinal cord module. The spinal cord module combines the motor commands with afferent feedback from the mechanical arm to generate motoneuron outputs controlling arm muscles for movements. **(B)** Setup for the center-out reaching tasks. The arm model simulates 2D reaching movements as a human subject performs with a robotic manipulandum. Reaching tasks are defined as moving the arm from the initial position at the center of the circle to one of target positions (sample target positions are shown by blue dots). For all simulations, the target reaching distance was fixed at 0.2 m and the reaching time was set to 1 sec. Angles **θ**_1_ and **θ**_2_ represent the shoulder and elbow joint angles, respectively, with respect to the vertical axis (gray lines).

The model considered in this study consists of three main modules: a brain module, a spinal cord module, and a biomechanical arm module (Figure 1A). The brain module has two parts: the cortex and basal ganglia (BG). The cortex includes the premotor cortex (PMC) whose outputs modulate the activity of primary motor cortex (M1), serving as inputs to the spinal cord module (motor program). The spinal cord module receives supraspinal motor commands and afferent feedbacks from muscles in the biomechanical arm module and forms motoneuron outputs. The biomechanical arm module takes inputs from motoneurons in the spinal cord module, activates the corresponding muscles to generate the arm movement to perform two-dimensional (2D) reaching tasks, and sends afferent feedback to the spinal cord module.

BG network receives inputs from prefrontal cortex (PFC), representing sensory cues, and PMC. In turn, the BG project to PMC thus forming a closed loop system. The role of BG is to activate a set of PMC neurons representing a behavioral response (action) appropriate to the sensory cue presented. It does so via reinforcement learning procedure that adjusts the weights of synaptic projections from PFC to BG. If the same sensory cue repeatedly evokes the same action for many trials, the association between the cue and the motor response get memorized and is stored in the cortex via emergence and potentiation of direct projections from PFC to PMC. Hereinafter we refer to this process as habituation.

The simulation of each trial is done in three stages. First, after the presentation of a sensory, resulting in activation of one or more PFC neurons, we calculate a steady state of the BG-PFC network, and use PFC firing rates to construct a motor program. Then, we use this motor program as an input to the neuro-mechanical arm model to simulate the movement. After that, we use the end point of the movement to evaluate if the movement was successful (reach the target) and to calculate the reward signal. Finally, we use the obtained reward to update the synaptic weights of PFC to BG projections based on the reinforcement learning rules. We describe the details of the model structure and mechanisms in following subsections.

### Biomechanical arm

**Figure 2:**
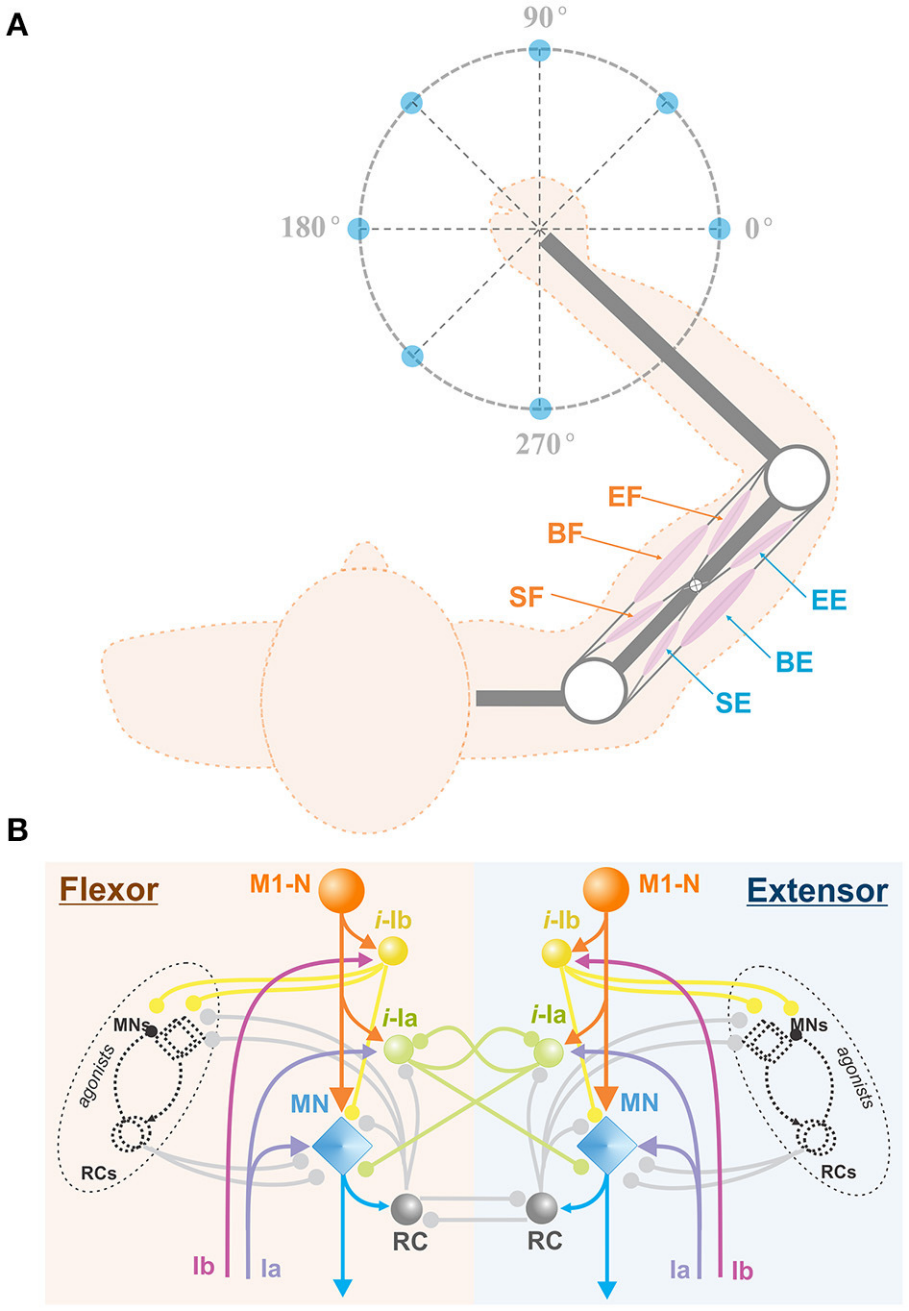
**Arm model and spinal cord network. (A)** Biomechanical arm model. The arm consists of 2 joints (white circles) at the shoulder and the elbow and 6 Hill-type muscles (pink ellipses), shoulder flexor (SF), bi-articular flexor (BF), elbow flexor (EF), shoulder extensor (SE), bi-articular extensor (BE), and elbow extensor (EE). This arm is designed to perform 2D reaching movements as described in Figure 1B. **(B)** Spinal cord network with afferent feedback. The spinal cord comprises interconnections of MNs, RCs, and interneurons. M1-Ns send motor command signals to MNs in the spinal cord, and MNs send muscle control signals to their corresponding arm muscles. The Ia and Ib inputs are the afferent feedback signals from the muscles. Interneurons take Ia and Ib inputs and modulate MN outputs. With RC connections, the spinal cord network mediates reflex responses. M1-N, Motor Cortex Neuron; *i*-la, la Interneuron; *i*-lb, lb Interneuron; RC, Renshaw Cell; MN, Motor Neuron; Ia, la afferent; Ib, lb afferent.

To simulate center-out reaching tasks, we previously designed an arm model simulating 2D center-out reaching movements (Figure 1B) (Teka et al., in press). The model (Figure 2A) consists of two rigid links connected by hinge joints (shoulder and elbow) actuated by six Hill-type muscles (Harischandra and Ekeberg, 2008). These include four single-joint muscles: the shoulder flexor (SF) and extensor (SE), the elbow flexor (EF) and extensor (EE), which control rotation of either the upper arm or forearm around the corresponding joint. The other two muscles, namely: bi-articular flexor (BF) and extensor (BE) are two-joint muscles that attached to both joints and simultaneously control movement around them. The arm movement depends on the combination of multiple muscle activations and is restricted to the horizontal plane. The dynamics of the arm motion is derived from the Lagrange equations, which take into account the Coriolis and centrifugal forces, joint viscoelastic forces, and muscle forces (Teka et al., in press). The proprioceptor afferent feedback from each muscle (Ia and Ib) projecting to the spinal cord was derived and modified from Prochazka (1999).

### Spinal cord network

The model of spinal cord (Teka et al., in press) comprises complex interconnections among motorneurons, interneurons, including Renshaw cells, Ia-, and Ib- inhibitory interneurons and correspondent afferent feedbacks (see Figure 2B). The spinal circuitry receives descending inputs from the motor cortex that activates corresponding motoneurons, which in turn drive arm movements via activation of muscles. Interneurons and Renshaw cells mediate interactions within spinal circuits and modulate cortical signals to the arm muscles. In addition, spinal reflexes, namely: stretch reflex, autogenic inhibition reflex, and recurrent inhibition of motoneurons, which play an important role in arm kinematics (Franklin and Wolpert, 2008), are incorporated into the model of spinal cord. The detailed description of the spinal circuitry can be found in previous publications (Franklin and Wolpert, 2008; Markin et al., 2016; Teka et al., in press).

### Basal ganglia network

**Figure 3:**
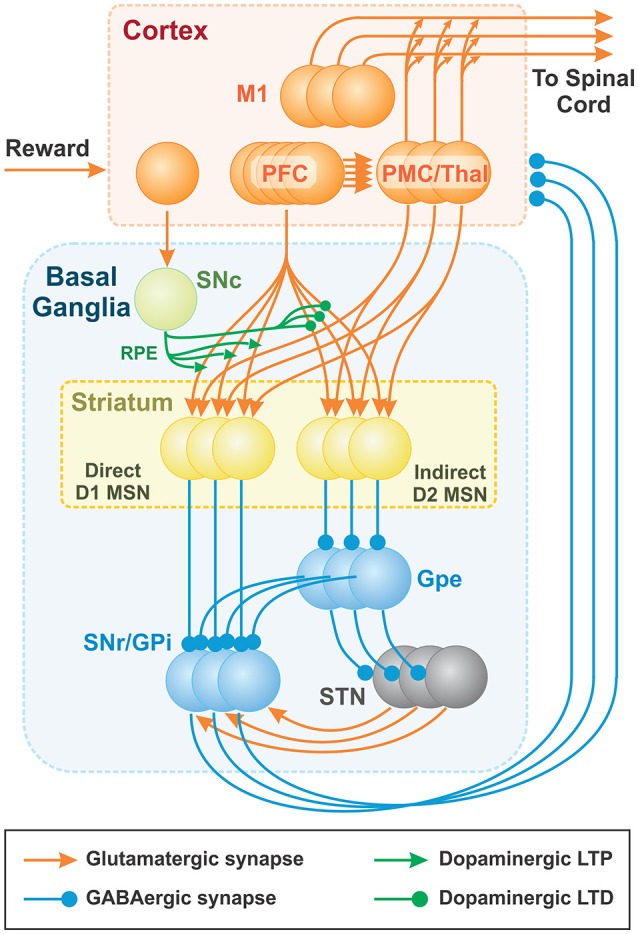
**Thalamocortical and Basal ganglia networks**. Each population consists of 100 non-spiking neurons (shown as 3 balls in each node), which correspond to 100 basic actions (see text for details). Basic actions are reaching movements of the arm from the initial position to one of 100 target positions. The PFC neurons represent “cues” and the PMC outputs define contributions of basic “actions” to the resulting motor program. The BG network creates the cue-action associations via the adjustment of PFC to MSN projections based on reinforcement learning mechanism. Dopaminergic SNc signal represents the reward prediction error (RPE). PFC (PreFrontal Cortex); M1 (Primary Motor Cortex); MSN (Medium Spiny Neuron); SNr (Substantia Nigra pars Reticulata); GPi (Globus Pallidus internal); GPe (Globus Pallidus external); SNc (Substantia Nigra pars Compacta); STN (SubThalamic Nucleus).

The brain module includes two major components: the cortex and the BG (Figure 3). The cortical component consists of the prefrontal cortex (PFC) and the premotor cortex (PMC). The PFC is comprised of neurons representing sensory stimuli (*cues*), and the PMC consists of neurons whose firing defines activation of different behavioral choices (*actions*). To prevent simultaneous activation of multiple cortical neurons, we incorporated relatively strong lateral inhibition in the PMC: i.e., each PMC neuron inhibits all other PMC neurons, such that strong activation of a few cells prevents other cells from firing. The resulting PMC network is multi-stable; the choice of an action may depend on the initial states of the neurons. See Discussion for some speculations on the network architecture that may provide lateral inhibition in the thalamocortical circuits.

The BG are composed of D1 and D2 MSNs, the GPe, the SNr/GPi, the STN, and the SNc. We combined the SNr and the GPi into the SNr/GPi as the integrated output of BG. Each BG node is implemented as a population of non-spiking neurons described in terms of their firing rates (see the complete mathematical description below). All populations contain the same number of neurons, each of which is correspondent to a single action. All neurons associated with the same action form cortico-striatal-thalamocortical loops that include two major pathways: (i) a direct pathway composed of D1 MSNs in the striatum and (ii) an indirect pathway, mediated by D2 MSNs. Specifically, in the direct pathway, input signals from the PFC and PMC pass through D1 MSNs directly to the SNr/GPi. In the indirect pathway, input signals from the PFC and PMC pass through D2 MSNs, the GPe, the STN and then the SNr/GPi. The final output signals of BG are relayed to the thalamus whose outputs feed back to the PMC to form parallel cortico-striatal-thalamocortical loops corresponding to different actions. The PFC, PMC, STN, and thalamus populations are composed of excitatory (glutamatergic) neurons, while striatal, GPe, and SNr/GPi populations are composed of inhibitory (GABAergic) neurons. The SNc consists of dopaminergic neurons.

To perform a motor task, such as reaching to a specific target in response to a specific cue, the BG network learns to associate the active sensory cue with appropriate motor actions through activation of one or more PMC neurons. After executing the action and gaining positive reward feedback, the BG associate the cue with the executed action through reinforcement learning mechanisms, based on a reward prediction error signal. Once cue-action associations are established, the BG can repeatedly select appropriate actions. Mechanistically, positive reinforcement on subsequent trials results in stronger activation of D1 MSNs directly inhibiting corresponding neurons in the SNr/GPi, which in turn disinhibit corresponding neurons in the thalamus that excite PMC neurons. However, when D2 MSNs are activated, they inhibit corresponding GPe neurons, which consequently leads to disinhibition of corresponding SNr/GPi neurons and inhibition of corresponding thalamic and PMC neurons. Therefore, the indirect pathway is instrumental in prevention of previously associated action execution in case of changing rules/conditions.

An exploratory mechanism is necessary to allow the BG to discover rewarding behaviors, which in turn enables cue-action associations to form. In our model, the BG exploration occurs stochastically. We implement exploratory mechanisms of BG model by incorporating stochastic fluctuations in the SNr/GPi population activity. Another source of BG exploration is multi-stability of the PMC network. Before each trial, we randomly set the initial states in the network. The activation probability of PMC neurons during decision making is defined by the BG network (PFC to D1 and D2 MSN connection weights), and by direct projections between PFC and PMC (see Figure 3).

#### Initial acquisition

Initial acquisition occurs through competition between the cortico-BG-thalamo-cortical loops, which depends on positively or negatively reinforced projections from PFC to MSNs. When the network initially selects a rewarding action by chance, the loop associated with that action gets advantage over other loops by LTP in the active D1-MSN and LTD in the active D2-MSN. If an action does not generate a reward, the corresponding cue-action association is weakened by LTD in the active D1-MSN and LTP in the active D2-MSN resulting from a negative RPE, thus reducing the probability for the same action to be selected on subsequent trials. Over many trials, acquisition occurs when the current PFC input becomes associated with the D1 MSNs that produce rewarding actions. In future trials, actions are selected and executed whenever the associated sensory PFC input is activated. In contrast, if an action results in non-rewarding outcomes, the corresponding sensory PFC input becomes associated with D2 MSNs corresponding to action suppression. Whenever the associated sensory PFC input is activated, non-rewarding actions are suppressed, which increases the probability of some other learned or spontaneous actions to be chosen under similar sensory conditions.

#### Reinforcement learning

Synaptic connections from the PFC to striatal MSN populations experience reward-based synaptic LTP and LTD in accordance with the implemented reinforcement learning rule. Striatal populations receive dopaminergic input from the SNc, which represents a trial-to-trial reward/punishment system in the model. The SNc in the model provides a RPE signal equal to the difference between the received and expected reward. We assume that cortical inputs to the SNc carry reward information derived from the performance of a motor action. In its turn, the SNc generates a differential reward signal, i.e., RPE, with the expected reward equal to the exponentially weighted average of rewards received during previous trials with the same sensory input. Whether an action is rewarded depends on the error between the task's target and the outcome of the executed action. In the model, an action is rewarded if the hand reaches within a preset distance from the target.

We do not explicitly model dopamine release by the SNc neurons. Instead, we assume that positive RPE corresponds to the increase in dopamine release, which to LTP of PFC projections to the active D1 MSNs and to LTD of the PFC connections to the active D2 MSNs. Conversely, a negative RPE corresponds to a dip in dopamine release, which potentiates synaptic connections from PFC to D2 MSNs and depress those to D1 MSNs. Therefore, if RPE is negative, active direct pathway connections are depressed, while active indirect pathway connections are potentiated to avoid the current cue-action pairing on subsequent trials. This mechanism was implemented in the model through varying synaptic connection weights between PFC neurons and MSNs of the direct and indirect pathways depending on the sign of the RPE signal.

#### Habituation

Habituation is the long-term process of developing habitual responses to frequently repeated sensory inputs. Piron et al. (2016) showed that well-learned behavioral responses remain intact even after the BG output is blocked by pharmacological suppression of the GPi activity. However, novel associations could not be learned under the same condition. This demonstrates that habitual cue-action associations are stored elsewhere and are not affected by blockade of BG output. Piron et al. suggested that the PFC is responsible for habit formation based on physiological data and computational models for habit learning (Atallah et al., 2007; Samejima and Doya, 2007; Graybiel, 2008). In our model, the cortex is represented by two populations, the PFC and PMC. In order to implement habituation, or cue-action association at the cortical level, we included a Hebbian learning mechanism in direct connections between PFC and PMC populations. Hebbian learning in the model represents the slower and more gradual acquisition of habits and the persistence of these habits over longer periods than reinforcement learning for BG-mediated cue-action associations. This mechanism allows the model to perform habitual action selection when the BG output was blocked (see section Disruption of reward based learning by GPi blockade).

#### Action selection

Plastic cortico-striatal projections from the PFC population to striatal D1 and D2 populations determine action selection in the BG. Previously learned and habituated cue-action associations allow the BG to select appropriate actions due to two mechanisms. First, previously reinforced BG-mediated cue-action associations, represented by potentiated connections from the PFC to D1 MSNs, activate direct pathways that correspond to more rewarding actions. Second, due to habituation process, sensory inputs directly cause increased activity of the corresponding PMC neurons. If currently activated sensory inputs do not have any associations, the system does not favor any response and equiprobably provides one of all possible actions given the current experimental context. Activated D1 MSNs inhibit corresponding SNr/GPi neurons, which in turn disinhibit corresponding PMC neurons that gate desirable actions. In contrast, activation of D2 MSNs lead to indirect disinhibition of the corresponding GPi neurons. Therefore, the state of cortico-striatal synaptic weights from the PFC to D1 and D2 MSNs and cortico-cortical projections from the PFC to the PMC permits the system to determine a preferred action.

#### Habit suppression and reversal learning

In the scenario where a cue-action association becomes invalid, the BG must be able to perform reversal learning, where the old cue-action association is suppressed and a new cue-action association is formed. The mechanism of suppression of preexisting associations depends chiefly on the D2 MSN indirect (NOGO) pathway. When the model performs the task that has previously been rewarded at the onset of a perturbation, the action does not produce a reward since it is perturbed. In this case, the RPE becomes negative. As a result, the PFC connections to active D1-MSNs get depressed, and the PFC connections to active D2-MSNs are potentiated. Increased activity in the D2-MSNs disinhibits the corresponding neuron in the SNr/GPi such that the action associated with that loop is suppressed. As such, D2-MSN activity can suppress the actions even if they were previously habituated. In this manner, the BG network suppresses the previous cue-action association and reverts to an exploratory mode, and a new cue-action association can be acquired as described above.

#### Mathematical model description

All neurons in all BG populations and the PMC are described based on a rate model of a neuron whose steady-state activation depends on the weighted sum of inputs and is described by a sigmoidal function as follows:

(1) $$\sigma (x)=\{\begin{array}{c} \;0\;,\;if\;x\leq 0\\ \tanh x,\;if\;x>0 \end{array}$$

where σ(*x*) is the normalized steady-state firing rate or the activity level and *x* is the sum of synaptic inputs to the neuron.

The instantaneous activity of the *i* th neuron in each population, *A*_*i*_, except PFC, adapts to the steady state with a time constant, τ = 1 *msec*, and is described by the following differential equation:

(2) $$\tau \frac{dA_{i}}{dt}=\sigma (I_{i})-A_{i}$$

where *I*_*i*_. is the total synaptic input to the cell. All populations representing the BG nuclei, except SNc, contain the same number of neurons equal to the number of basic actions *N*_*a*_ (see below), such that each neuron in each BG nucleus corresponds to one basic action. The SNc produces a scalar dopaminergic reward signal that codes the RPE (see below).

The PFC sensory neurons, representing all possible sensory cues project to all D1 and D2 MSNs. Initial connection weights between PFC neurons and striatal MSNs are randomly set between 0 and 0.001 and then vary trial-to-trial based on the reinforcement learning rule formulated below. Initial conditions for activities of D1 MSNs, D2 MSNs, and PMC neurons are uniformly randomly distributed between 0 and 0.1.

As mentioned, the spiny neurons (both D1 and D2 MSNs) receive synaptic inputs from all PFC neurons and from PMC neurons associated with the same action. Accordingly, the activity level of each D1 MSN, $D_{i}^{1}$. is driven by Equation (2) with the synaptic input, $I_{i}^{D1}$, as follows.

(3) $$I_{i}^{D1}=\sum_{j\;=\;1}^{N_{c}}W_{ji}^{1}\cdot C_{j}+w_{a}^{1}\cdot M_{i}$$

where $\;W_{ji}^{1}$ is the connection weight between PFC neuron corresponding to cue *j* and D1-MSN *i*, which varies trial-to-trial; *C*_*j*_ is the activity level of the PFC neuron *j*; $w_{a}^{1}=2$ is the connection weight between PMC neuron *i* and D1-MSN *i* corresponding to the same action; *M*_*i*_ is the activity level of PMC neuron *i*.

Similarly, the synaptic input to the D2-MSN *i*, $I_{i}^{D2}$, is

(4) $$I_{i}^{D2}=\sum_{j\;=\;1}^{N_{c}}W_{ji}^{2}\cdot C_{j}+w_{a}^{2}\cdot M_{i}$$

where $\;W_{ji}^{2}$ is the connection weight between PFC neuron *j* and D2-MSN *i*, which varies trial-to-trial; $w_{a}^{2}$ = 2 is the connection weight between PMC neuron *i* and D2-MSN *i*.

The synaptic input to *i* th GPe neuron, $\;I_{i}^{GPe}$ is defined as:

(5) $$I_{i}^{GPe}=Dr_{GPe}-w_{d2}\cdot D_{i}^{2}$$

where *Dr*_*GPe*_ = 2 is the tonic drive to GPe neurons; *w*_*d*2_ = 2 is the weight of the inhibitory connection between D2-MSN *i* and GPe neuron *i*, and $D_{i}^{2}$ is the activity of the ith D2-MSN.

The synaptic input to *i* th STN neuron $I_{i}^{STN}$ is defined as:

(6) $$I_{i}^{STN}=Dr_{STN}-w_{GPe}\cdot GPe_{i}$$

Where *Dr*_*STN*_ is the tonic drive to STN neurons; *w*_*GPe*_ = 1 is the weight of the inhibitory connection between GPe neuron *i* and STN neuron *i*; *GPe*_*i*_ is the activity of the *i* th GPe.

The synaptic input to the *i* th GPi neuron, $I_{i}^{GPi}$ is defined as:

(7) $$I_{i}^{GPi}=Dr_{GPi}-w_{d1}\cdot D_{i}^{1}+w_{STN}\cdot STN_{i}$$

where *Dr*_*GPi*_ = 0.2 is the tonic drive to GPi neurons; *w*_*d*1_ = 2 is the weight of the inhibitory connection between direct pathway MSN *i* and GPi neuron *i*; *w*_*STN*_ = 1 is the weight of the excitatory connection between STN neuron *i* and GPi neuron *i*; and *STN*_*i*_ is the activity of the *i* th STN neuron.

The synaptic input to the *i* th PMC neuron, $\;I_{i}^{M}$ is defined as:

(8) $$\begin{array}{l} I_{i}^{M}=Dr_{M}+\sum_{j\;=\;1}^{N_{c}}W_{ji}^{CM}\cdot C_{j}-w_{gpi}\cdot GPi_{i}\\ \;-\;\sum_{j\;\neq \;i}^{N_{a}}w_{inh}^{M}\cdot M_{i} \end{array}$$

where *Dr*_*M*_ = 1.3 is the tonic drive to PMC neurons; $\;W_{ji}^{CM}$ is the connection weight between PFC neuron *j* and PMC neuron *i*; *w*_*gpi*_ = 1.8 is the weight of the inhibitory connection between GPi neuron *i* and PMC neuron *i*, $w_{inh}^{M}=1.7$ is the weight of inhibitory connection from *j* th to *i* th PMC neuron, and *M*_*j*_ is the activity of the *j* th PMC neuron.

We do not explicitly model the Thalamus as a neuronal population. The Thalamus directly excites the PMC, and we do not consider any dynamics in or modulation of these projections. For simplicity, these two entities have been combined and modeled as a single PMC population that receives inhibition from the GPi.

Reinforcement learning, i.e., the trial-to-trial changes in the striatal connection weights in the model, is implemented as follows:

(9) $$\Delta W_{ji}^{1}=\lambda_{1}\cdot SNc\cdot C_{j}\cdot D_{i}^{1}-d_{w}\cdot W_{ji}^{1}$$

(10) $$\Delta W_{ji}^{2}=-\lambda_{2}\cdot SNc\cdot C_{j}\cdot D_{i}^{2}-d_{w}\cdot W_{ji}^{2}$$

where $\;\Delta W_{ji}^{1}$ and $\Delta W_{ji}^{2}$ are the changes in synaptic weights between PFC neuron *j* and D1- and D2-MSNs *j*, respectively; λ_1_ = 0.6 and λ_2_ = 0.6 are the learning rates for the PFC to D1 (direct pathway) and PFC to D2 synaptic weights, respectively; *SNc* is the SNc dopaminergic reward/punishment signal, and *d*_*w*_ = 0.02 is a degradation rate (synaptic scaling parameter). Weights $\;W_{ji}^{1}$ and $\;W_{ji}^{2}$ are accepted non-negative and set to 0, otherwise.

Formulae (9, 10) represent the idea that the actions that led to a positive reward signal from SNc get reinforced via incremented $W_{ji}^{1}$ weights, and those that led to a negative reward signal from SNc get suppressed via incremented $W_{ji}^{2}$ weights.

The SNc dopaminergic reward/punishment signal as a reward prediction error (RPE) which is equal to the difference between the reward for the current trial and the expected reward as follows:

(11) $$SNc=R-Re_{j}$$

where *Re*_*j*_ is the expected reward for cue *j*, calculated as an exponentially weighted average of rewards received during previous trials (see below), and *R* is the reward for the current trial.

The reward is determined by whether the final position when the performed reaching movement is within the target spot, defined as a circle around the target position:

(12) $$R=\Theta \left({\text{D}}_{max}-D_{err}\right)$$

where Θ(·) is Heaviside step function, *D*_*err*_ is the distance error between the target position and the actual final position of the arm. The radius of the rewarding spot, *D*_*max*_, varies depending on the experimental setup simulated.

The expected reward is updated as an exponential moving average of previously obtained rewards:

(13) $$Re_{j}^{k\;+\;1}=(1-\alpha_{R})Re_{j}^{k}+\alpha_{R}R^{k}$$

where *k* is a trial number, α_*R*_ = 0.15 discounts previous reward observations in ~6 trials, and *R*^*k*^ is the reward obtained on *k*th trial.

The cortical Hebbian learning rule is implemented as follows:

(14) $$\Delta W_{ji}^{CM}=\lambda_{CM}\cdot C_{j}\cdot M_{i}-d_{CM}\cdot W_{ji}^{CM}$$

where $\Delta W_{ji}^{CM}$ is the change in the synaptic weight between PFC neuron *j* and PMC neuron *i*; λ_*CM*_ = 0.001 is the learning rate; *d*_*CM*_ = 0.001 is the connection decay rate.

When coupling the BG output to the motor control system, we assume that each PMC corresponds to one basic action represented as a motor program. Each PMC neuron has connections to 6 populations in the primary motor cortex which, in turn, project to 6 corresponding motoneuronal populations in the spinal cord, each of which controls a muscle in the mechanical arm. In our model, a basic action is defined as a reaching movement in a given direction from a preset initial position with fixed distance and reaching time. We arbitrarily chose *N*_*a*_ = 100 uniformly distributed directions in 0° ~ 360° angles as basic movements. Accordingly, the set of all basic actions consisted of *N*_*a*_ vector functions representing temporal patterns of motocortical drives actuating the spinal cord motor circuits:

(15) $${\left\{{\vec{Mdr}}_{k}\left(t\right)\right\}}_{k\;=\;1}^{\;N_{a}}$$

where each ${\vec{Mdr}}_{k}\left(t\right)$ has 6 time-dependent components serving as inputs to corresponding spinal circuits, each associated with a biomechanical arm muscle. For each reaching movement corresponding to a different direction, these components were calculated by solving the inverse problem based on a predefined arm trajectory as described in Teka et al. (in press).

The BG activity and the synaptic weights of PFC projections to the PMC population determine the PMC population activity, which consists of *N*_*a*_ action-associated neurons. The resulting motor program was constructed as a linear combination of all possible actions (Equation 15) with coefficients defined by the activity level of PMC neurons *M*_*k*_:

(16) $$\vec{Mdr}\left(t\right)={\vec{Mdr}}_{0}+C_{M}\sum_{k=1}^{N_{a}}M_{k}\left({\vec{Mdr}}_{k}\left(t\right)-{\vec{Mdr}}_{k}\left(0\right)\right).$$

where *C*_*M*_ = 1.3 is a scaling coefficient. The initial value ${\vec{Mdr}}_{0}={\vec{Mdr}}_{k}\left(0\right)$ is the same for all motor programs and is defined by posture of the arm at the initial position. In the form of Equation (16) the generated motor program $\vec{Mdr}\left(t\right)$ has the same initial value regardless of PMC activity to preserve the arm posture at the initial point, while the dynamic components of motocortical drives are defined by the activity level of PMC neurons associated with different actions.

Motor program (Equation 16) is used as an input to the neuro-mechanical model of the arm described in details in Teka et al. (in press). After the end position of the movement is computed, a small random perturbation representing motor noise is added. This perturbation is modeled as an independent Gaussian random variable with zero mean and 5 mm standard deviation.

### Simulation of experimental data

To validate the model, we simulated the results of several experimental studies related to non-error based motor adaptation as it is believed to be mediated only by the BG (see Discussion).

#### Motor adaptation during force field perturbation

Ahmadi-Pajouh et al. (2012) introduced an experimental setup where human subjects performed reaching tasks and adapted to a standard curl force field as a perturbation. In experiments, subjects held and moved the handle of a robotic manipulandum without direct visual feedback, and thus they could not see hand or handle movements directly but could see only the starting point of the handle and a target point to reach on a projected screen above the plane of a hand. We consider this an example of non-error based learning because although subjects could see the endpoint, the error information could not be directly used to correct movements due to the strong complex perturbation. Each experiment session had three sequential phases in 9 blocks: *baseline* when the curl force field was null (3 blocks), *adaptation* when the curl force field in 45°Clockwise (CW) or 225°Counter clockwise (CCW) was applied to the manipulandum (3 blocks), and *washout* when the applied force field was removed (3 blocks). Subjects were divided into 3 groups: CW group, CCW group, and control group.

To simulate force field perturbations, we modified the reaching model to apply external forces at the moving endpoint of the biomechanical arm (hand) (**Figures 5A,B**). The magnitude of the force depended on the hand's velocity, while its direction was perpendicular to the hand's moving direction. Our implementation of force field perturbations emulates experimental studies where velocity dependent force fields were used to perturb normal reaching movements (Shadmehr and Mussa-Ivaldi, 1994; Scheidt et al., 2000; Franklin et al., 2003; Ahmadi-Pajouh et al., 2012).

Perturbations are introduced in the biomechanical arm model as two perturbation torques: *TS*_*F*_ (applied at the shoulder joint) and *TE*_*F*_ (applied to the elbow joint), in addition to existing torques:

(17) $$TS=TS_{C}+TS_{NC}+TS_{F}$$

(18) $$TE=TE_{C}+TE_{NC}+TE_{F}$$

where *TS* and *TE* are the total torques at the shoulder and elbow joints, respectively; *TS*_*C*_ and *TE*_*C*_ are the torques due to conservative forces acting at the two joints; *TS*_*NC*_ and *TE*_*NC*_ are the torques due to non-conservative forces (joint frictional forces during movement, joint frictional forces at their motion range limits, and muscle forces) acting at the two joints; *TS*_*F*_ and *TE*_*F*_ are the torques due to the force field perturbation. (See Teka et al., in press for a complete set of equations of motion).

The perturbation torques at the two joints are calculated as z-components of the vector products of the perturbation force and corresponding moment arm vectors:

(19) $$\text{T}S_{F}=r_{Sx}\cdot F_{y}-r_{Sy}\cdot F_{x}$$

(20) $$\text{T}E_{F}=r_{Ex}\cdot F_{y}-r_{Ey}\cdot F_{x}$$

where *r*_*Sx*_ and *r*_*Sy*_ are the *x* and *y* components of the moment arm vector of the perturbation force relative to the shoulder joint in the horizontal Cartesian plane of motion; similarly, *r*_*Ex*_ and *r*_*Ey*_ are the components of the moment arm relative to the elbow joint; and *F*_*x*_ and *F*_*y*_ are the *x* and *y* vector components of the perturbation force.

The moment arms at the shoulder joint are given by:

(21) $$r_{Sx}=L_{1}\cdot sin\left(\theta_{1}\right)+r_{Ex}$$

(22) $$r_{Sy}=L_{1}\cdot cos\left(\theta_{1}\right)+r_{Ey}$$

where *L*_1_ is the length of the upper arm segment of the limb; and θ_1_ is the angle between the upper arm and the y-axis of the plane of motion.

The components of the moment arm vector relative to the elbow joint are given by:

(23) $$r_{Ex}=L_{2}\cdot sin\left(\theta_{2}\right)$$

(24) $$r_{Ey}=L_{2}\cdot cos\left(\theta_{2}\right)$$

where *L*_2_ is the length of forearm segment; and θ_2_ is the angle between the forearm and the y-axis of the plane of motion (see Figure 1).

Finally, *x* and *y* vector components of the perturbation force are given by:

(25) $$F_{x}=-\alpha \cdot v_{y}$$

(26) $$F_{y}=\alpha \cdot v_{x}$$

where α is a force scaling coefficient used to modify the direction and strength of the force field; and *v*_*x*_ and *v*_*y*_ are the wrist's *x* and *y* velocity components, respectively. If α < 0, the force field is in the CW direction, and if α > 0, the force field is in the CCW direction.

*v*_*x*_ and *v*_*y*_ are

(27) $$v_{x}=L_{1}\cdot {\dot{\theta }}_{S}\cdot cos\left(\theta_{1}\right)+L_{2}\cdot {\dot{\theta }}_{E}\cdot cos\left(\theta_{2}\right)$$

(28) $$v_{y}=-L_{1}\cdot {\dot{\theta }}_{S}\cdot sin\left(\theta_{1}\right)-L_{2}\cdot {\dot{\theta }}_{E}\cdot sin\left(\theta_{2}\right)$$

where ${\dot{\theta }}_{S}$ and ${\dot{\theta }}_{E}$ are the angular velocities of the shoulder and elbow joints, respectively.

To reproduce Ahmadi-Pajouh et al.'s experimental setup, we used a single cue (represented by a single neuron in the PFC) in response to which a movement to the north target was rewarded (90° in Figure 1B). Each experiment session had total 1,000 trials in 3 phases: baseline (300 trials), adaption (350 trials), and washout (350 trials). The simulated force field perturbation was introduced only at trial 301 and removed at trial 651 for the adaptation phase. For CW and CCW groups, we performed two sets of simulations, one with a force field in the CW (α = −2) direction and the other in the CCW (α = 2) direction. A total of 16 simulated sessions were averaged by trial for the CW or CCW groups. The rewarding spot radius, *D*_*max*_ was 8 cm.

#### Motor adaptation during visual rotation perturbation

The experiment by Galea et al. (2011) was to measure the quick “shoot” reaching performance of human subjects over a short 0.1 m distance. Right-handed subjects were asked to move a digitizing pen very quickly from an initial center position to a target position located in one of 8 directions. The motion of this pen was displayed as the motion of a cursor on a computer screen. The purpose of this quick shooting was to suppress the engagement of possible online error correction mechanisms during reaching via visual feedback when a visual perturbation was applied. The visual perturbation was defined as a visual rotation of reaching trajectories displayed on the screen by 30° CCW. The experimental protocol included epochs (one epoch consisted of 8 consecutive trials) with and without visual rotation. Initially there was no perturbation for 24 epochs (Pre 1 and 2), then visual rotation was applied for 25 epochs (Adapt 1), and visual rotation returned to the initial state in Pre 1 and 2 for 12 epochs.

To simulate this experimental setup, we used a stronger (90°) CCW transformation as the effect of visual rotation. During the pre-learning process, the presented sensory cue (C1) was associated with the rewarding target action (T1) located at the east (0°) for 700 trials. In the main simulation, T1 was kept as a rewarding target for initial 24 trials, and then another target action (T2) located at the north (90°) (to mimic a 90°CCW visual rotation) was rewarded for the next 25 trials. The rewarding target returned back to T1 for the last 12 trials. Our goal here was to simulate motor adaptation by the BG during visual perturbations, so only the control group data (sham, black lines in **Figure 6A**) was used for comparison purposes. The rewarding spot radius, *D*_*max*_ was 7 cm.

#### Effects of the BG related diseases on motor adaptation

To test how the BG related diseases such as Huntington's (HD) and Parkinson's Diseases (PD) affect non-error based learning via the BG, Gutierrez-Garralda et al. (2013) proposed an experimental setup, where subjects were asked to throw a ball to a fixed target with visual perturbation. During the experiment, a dove prism was used which horizontally reversed the visual field and shifted the target's position. Under the effect of this visual perturbation, the error sign was reversed, and therefore when subjects missed the actual target to the right, they observed the error to the left of the perceived target (**Figure 7A**, trial 1) and corrected it by throwing further to the right (**Figure 7A**, trial 2), thereby increasing the magnitude of the error distance. However, to reduce the error, subjects had to correct by throwing further to the left (**Figure 7A**, trial 3). Therefore, the authors concluded that the error-based movement correction was not possible. Three subject groups participated in this experiment: the PD group, the HD group, and the healthy control group.

To mimic this setup with our model, we used the reaching movements instead of throwing. We used two different sensory cues, C1 and C2, and two reaching target actions T1 (to west) and T2 (to east). Via the pre-learning session, habitual associations for both cues (C1-T1 and C2-T2) were formed over 700 trials. During the simulated experiment, the T1 position corresponds to the actual target position, and the T2 position corresponds to the perceived, distorted target position as observed during the visual perturbation phase. For the first 25 trials of the simulation, only C1 was activated, and reward was given for reaching the T1 position. Then for trials 26 to 50, the activated cue switched from C1 to C2, and the reward was still given for reaching the T1 position. This setup works as if a subject throws a ball to the perceived, distorted target position and thus gets no reward. To reach the T1 position during the perturbation phase, C2 already associated with T2 needs to get associated with T1 via reversal learning. Therefore, a new cue-action association (C2-T1) should be formed to correct errors. Finally, in the last 25 trials the activated cue got back to C1 rewarding for reaching the T1 position. The rewarding spot radius, *D*_*max*_ was 8 cm.

The activity of all D2 MSNs was reduced by 90% to mimic the HD condition, which, as we assume, results in D2-MSN neurodegeneration. For the PD condition, which manifests itself by dopamine deficiency, we reduced the learning rates λ_1_, λ_2_ (see Equations 9, 10 in section Mathematical Model Description) in D1 and D2 MSNs by 90%.

#### Disruption of reward based learning by GPi blockade

To study the effect of pharmacological blockade of the BG output on decision making in different conditions, Piron et al. (2016) used the following experimental settings. Two monkeys were trained over a long period to perform decision-making tasks by selecting one of 4 virtual square buttons on a screen. From movement standpoint, these virtual buttons represent 4 different directions: north, south, east and west with respect to the center of a board (**Figure 8A**). Each monkey moved one of its hands on the board and the tracked hand position was displayed as a cursor movement on the screen. Button selection was done by moving the cursor from the initial center position inside a square representing the target button and keeping the hand inside this square for a while. Experiments were divided into the routine condition (RC) and the novelty condition (NC). In both conditions, visual cues were two virtual buttons highlighted with two different figures. The choice of two highlighted buttons among four buttons was random on every trial. Selection of one of two highlighted buttons was rewarded with different reward probabilities depending on the figure displayed: one with *P* = 0.75 and the other with *P* = 0.25. Specifically, when a monkey selected one of the highlighted buttons, it had a chance to get a treat, and whether the reward was given was based on the reward probability for the chosen figure (e.g., choosing the cross was rewarded in 75% of cases and the triangle in 25% of cases, see **Figure 8A**). Thus, selecting one of the highlighted buttons and remembering which figure has higher rewarding chance are important to receive reward since positions randomly change. During the preliminary training period, the monkeys were shown two figures, F1 and F2, on the buttons and learned to select F1, independent of its position, as the button with F1 was rewarded with greater (75 vs. 25%) probability). During the experiment, in the RC blocks, the highlighted buttons had the same figures, F1 and F2, which the monkey already learned. In the NC, the set of figures was changed to F3 and F4 which were clearly different from F1 and F2, and thus monkeys no longer knew which figure has higher reward probability and had to learn to choose the more rewarding figure.

To block the BG output, they injected muscimol (GABA_A_ receptor agonist) through the surgically pre-inserted cannula into the GPi, the BG output nucleus. In the control case, saline was injected into the GPi instead of muscimol. Per experiment, monkeys performed a session of 250 trials in which the RC and the NC were alternated every 10 trials. After muscimol injection, monkeys continued to select the more rewarding figure in the RC, but failed to learn in the NC.

To mimic experiments from Piron et al., we used 24 cues: C1–C12 each representing an ordered pair of two highlighted buttons for the RC and C13–C24 each corresponding to a new pair of buttons for the NC. Each highlighted button was represented by a pair of features: figure shape and figure position (e.g., the yellow cross on the north button or the yellow triangle on the west button, see **Figure 8A**). Twelve cues are sufficient to account for all possible combinations of positions and figures in this task. In each trial, one of 12 cues is randomly activated in RC or NC conditions.

When a NC cue is activated, there is no initial figure preference, and highlighted buttons with different figures are pressed with equal probability. Eventually, after sufficiently many trials, the NC cue is expected to associate with actions representing reaching movements to the button with higher reward probability.

For the RC, there are habitual associations between cues C1–C12 and the actions corresponding to reaching the button containing the more rewarding figure for that cue (having 75% reward probability). There are also habitual associations between each cue and its less rewarding (having 25% reward probability) action, but with smaller connection weights as those actions got reinforced a lot less often during initial training. For the NC cues we also have associations for both optimal and non-optimal actions, but their strengths do not correlate with the figures on the buttons. So, in the NC the selection of different figures is initially equiprobable.

To simulate the GPi blockade by muscimol injection, we suppressed 100% of the GPi output. Matching the Piron et al. setup, the RC and NC were alternated block by block in a session per experiment. In the model if the actual end-point by a reaching movement was within a circular target button with 0.2 m diameter, the button was considered as selected. The success rate was defined as the number of trials in which the most rewarding target of the two buttons was selected over the total number of trials in which any button was selected. All end reaching points outside of any button and to buttons, not corresponding to the current cue, were considered as errors, and were excluded from the success rate calculation across 20 simulations. The rewarding spot radius, *D*_*max*_ was 10 cm.

### Modeling environment

The model was implemented in C++, and simulated results were processed and visualized in Matlab.

## Results

### Model performance

**Figure 4:**
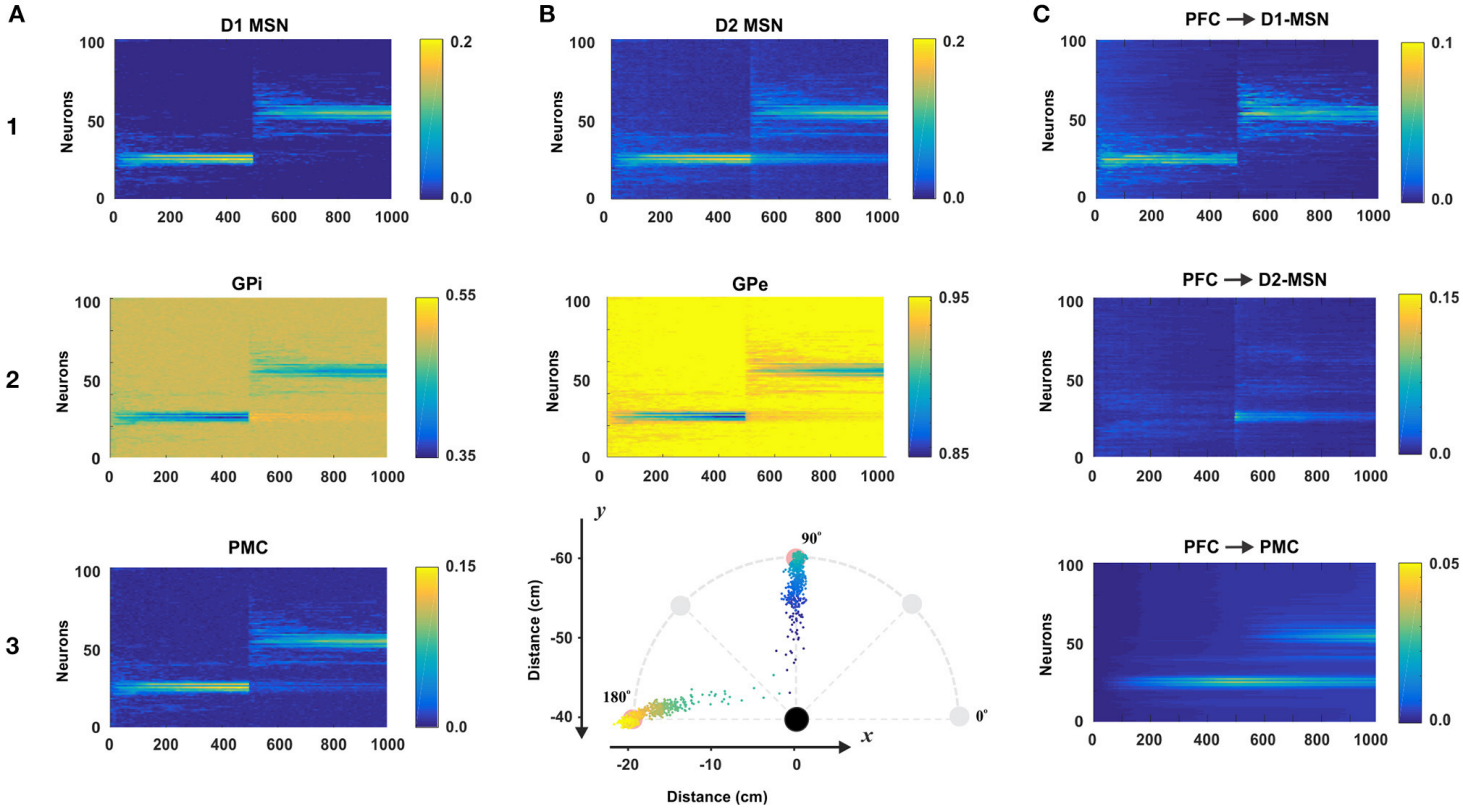
**Model performance**. All plots are averages of 100 simulations each run for 1,000 reaching trials. In all simulations, only one sensory cue was activated. During first 500 trials the rewarding spot was within 10 cm distance from the north target (at 90° on **B3**). After 500 trials the target was switched to the west (180° on **B3**). The first 500 trials comprise the acquisition phase in which the BG create an initial cue-action association via reinforcement learning. During last 500 trials, the BG suppress old no longer desirable cue-associations and explore for new ones to get larger rewards. Thus, the active cue associates with a new reaching direction. Total 100 experiment sessions were averaged by trial. (**A1–3** and **B1,B2**) The raster plots of BG nodes: D1-MSN, GPi, PMC, D2-MSN, and GPe. **(B3)** Mean reaching positions across 100 session (colored dots). For the first 500 trials, dots are headed for the north (90°) and for the last 500 trials, dots are headed for the west (180°). **(C)** The dynamics of the weights of connections from the PFC neuron representing the active cue to D1-, D2-MSNs, and PMC neurons.

To evaluate the role of each nucleus within the BG model, we simulated a simple reaching task over 1,000 trials. Specifically, we introduced one sensory cue (C1) and two target actions (T1 and T2) which corresponded to reaching toward north (T1) and west (T2) directions at 10 cm distance (to the points in 90° and 180°, respectively; see Figure 4B3). For the first 500 trials of the simulation, target action T1 was rewarded. For the second set of 500 trials, the rewarded target switched from T1 to T2. In total, 100 simulated experiments were averaged by trial. Figure 4 depicts these results with multiple nuclei outputs and connection weights.

#### BG nuclei activity during initial acquisition and reversal learning

For the first 500 trials, the model was supposed to learn to reach to the rewarding spot T1. To do so, the BG needed to activate appropriate neurons in the PMC to construct an action that maximize reward. In Figure 4A3 we can see that at the beginning of the simulation, PMC neurons were randomly activated producing movements far from the target. In this initial exploring period the BG randomly search for rewarding actions. Within 100 trials, the distribution of activated PMC neurons narrows down to the set of neurons having indices close to 25, whose activation results in a movement ending within the rewarding spot. In subsequent trials 100–500 we can see gradual increase in the activation magnitude of the PMC neurons due to potentiating direct PFC to PMC projections underlying habituation (see below). Since activated D1 MSNs inhibit corresponding GPi neurons, we can see activity in the GPi output with inverse power to the activity of D1 MSNs. Similarly, GPi neurons disinhibit corresponding PMC neurons which in turn excite D1 and D2 MSNs. So, similar activity distributions are seen in D1 and D2 MSNs and PMC neurons (Figures 4A1–A3).

During trials 500–1,000, the rewarded target action was switched from T1 to T2. Thus, the BG had to associate the cue presented with a new target. In the direct pathway, the pattern of D1 MSN activity in the second 500 trials is similar to the one in the first 500 trials (Figure 4A1): there is an initial exploring period followed by gradually narrowing distribution of activated neurons, and gradual increase in their activation magnitude. Unlike the activity of D1 MSNs, the activity of D2 MSNs after 500 trials has two distinct modes (Figure 4B1): one around neuron No. 50 for the new target T1 and the other around neuron No. 25 for the old target T1. This way, D2 MSNs in the indirect pathway play a key role in suppressing PMC neurons corresponding to the previously habituated but no longer relevant responses. When a D1 MSN competes with the corresponding D2-MSN with similar activity levels, the D1-MSN always wins due to the higher weight of connection to the corresponding GPi neuron. However, when the activity level of a D1 MSN is relatively low or close to zero, the corresponding D2 MSN with non-zero activity level wins in competition. In the indirect pathway, activated D2 MSNs inhibit corresponding GPe neurons, the inhibited GPe neurons inhibit STN, which excite corresponding GPi neurons, and GPi neurons (receiving competing inhibition from D1 and excitation from STN) inhibit corresponding PMC neurons. Based on this mechanism previously acquired and habituated cue-action associations get suppressed.

#### Connection weights during acquisition and learning

The connection weights from the PFC to D1-MSNs are subject to reinforcement learning for cue-action associations. As we can see in Figure 4C1, the weight dynamics of PFC->D1-MSN is similar to the outputs of D1-MSNs shown in Figure 4A1. For the first 500 trials, PFC->D1-MSN weights around neuron 25 were increased, and for the second 500 trials, the weights around neuron 25 immediately vanished, and the weights around neuron 50 were increased. In contrast, the PFC->D2-MSN weights nearby neuron 50 sparked immediately after trial 500 and gradually decreased in the second 500 trials (Figure 4C2). This clearly demonstrates the role of plastic PFC->D2-MSN projections in suppression of undesired habituated responses. With no learning in D2-MSN population, the developed habit to respond to the cue by reaching to the old target prevents the system from exploring new options, and the model continues to select no longer relevant action. In these conditions, the reversal learning becomes impossible or significantly impaired.

PFC->PMC weights (Figure 4C3) represent habitual cue-action associations in the cortex formed by a Hebbian learning rule. The weights near neuron 25 gradually increased for the first 500 trials and gradually degraded for the second 500 trials because the corresponding PMC neurons were no longer repeatedly activated. Instead, the weights around neuron 50 gradually increased for the second 500 trials. Because of this, we can see two wide strips around neurons No. 25 and No. 50 for the second 500 trials though the former is decreasing and the latter is increasing (Figure 4C3) as the new habit replaces the old one.

### Comparisons with experimental data

#### Motor adaptation during force field perturbation

**Figure 5:**
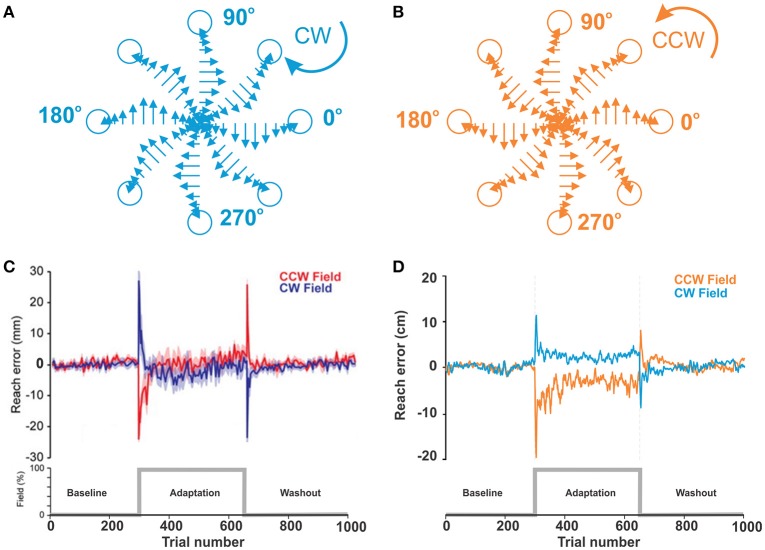
**Motor adaptation during force field perturbations. (A,B)** Force field applied to the biomechanical arm during reaching movements in 8 directions. Force magnitude is velocity dependent and is applied perpendicular to the direction of movement CW or CCW. **(C)** Reproduced from Ahmadi-Pajouh et al. (2012) with permission. Performance during reaching movements. The plot shows a measure of error in millimeters during reaching with force field perturbation by subjects in the CW and CCW groups. **(D)** Force-field perturbation simulated in the model using the same protocol. The plot shows reach errors in centimeters.

As described in Methods, in force-field perturbation experiments, human subjects moved the handle of a manipulandum to a target at 135°, 14 cm distance from the center position in a 2D plane with and without curl force applied to the handle. Figure 5C shows the experimental results. In the baseline phase without any perturbation, reach errors were relatively low within ±2 ~ 3 mm. When the curl force field was imposed at the trial 300, reach errors surged up to 30 mm with the CW field or—25 mm with the CCW field and decreased to near zero level roughly within 25 trials, though error fluctuations were larger than those in the baseline phase. In the aftereffect phase, the curve shape was similar as in the perturbation phase, but peak error directions were opposite and adaptation periods after peaks were shorter.

Figure 5D shows the results from our simulation of qualitatively similar conditions (see the figure caption for details). With the model, we simulated reaching movement to the target at the north (90°), 20 cm distance from the center. Movements within 8 cm distance from the target were rewarded. In the perturbation phase, average error peaks were 10 cm with the CW field and −20 cm with the CCW field. Then, reaching errors quickly adapted to the levels below the radius of the rewarding spot during approx. the same number of trials as in the experiments.

#### Motor adaptation during visual rotation perturbation

**Figure 6:**
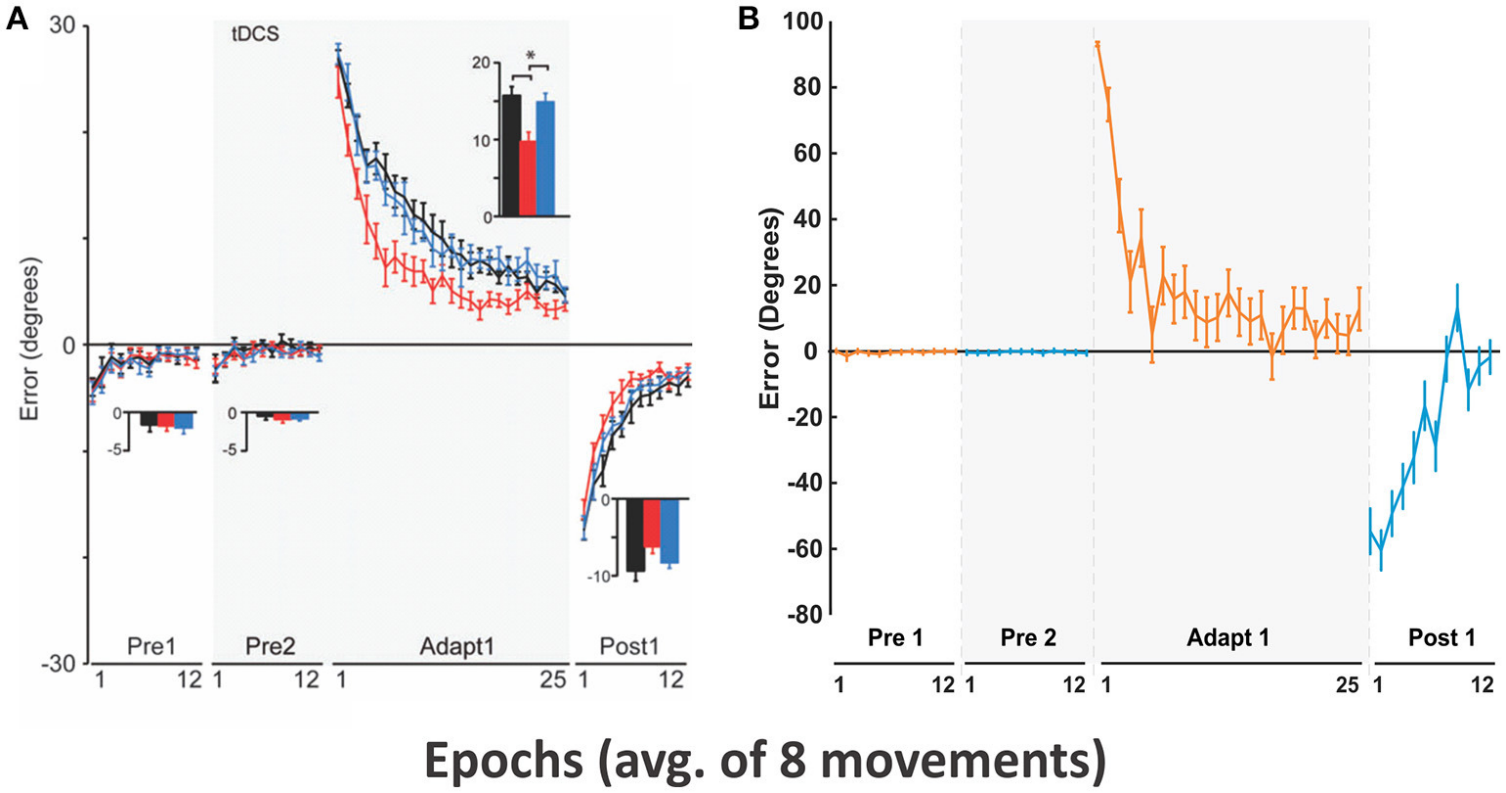
**Motor adaptation during visual rotation perturbation. (A)** Reproduced from Galea et al. (2011) with permission. End point error (in degrees) are shown during baseline (Pre 1 and 2), adaptation (Adapt 1), and post-adaptation (Post 1). Only conditions corresponding to the control group (black lines) were used for subsequent model simulation. In Adapt 1, subjects gradually adapted to the visual perturbation to reduce the errors (black line). In Post 1, aftereffects of motor adaptation from Adapt 1 gradually decreased in favor of error reduction. **(B)** Simulated results with our model.

Our simulation was aimed to represent results from the control group (see sham, black lines in Figures 6A,B). The experimental settings and our approach to mimic them in the model are described in Methods.

In the Pre 1 and Pre 2 phases, subjects' performance showing fairly straight hand trajectories was close to targets in 8 directions (Figure 6A) and average errors were low (see Pre1 and Pre2 in Figure 6B). In the Adapt 1 phase, when the visual rotation by CCW 30° was applied, subjects gradually adapted thus reducing errors from 30° to 5° in 25 epochs. In the Post 1 phase, errors in an opposite direction appeared up to the aftereffect of visuomotor adaption occurred in the Adapt 1 phase. However, subjects adapted to the normal conditions slightly faster (within 12 epochs) after the screen was rotated back.

Figure 6B shows that the model gradually adapted from initial 90° errors to 10° errors within 25 epochs. In the Post 1 phase, the direction of the errors was inverted to the negative and absolute errors gradually decreased to 0° with fluctuation. The overall results are qualitatively similar to the results of Galea et al. exhibiting comparable time scale for the adaptation.

#### Effects of the BG related diseases on non-error based learning

**Figure 7:**
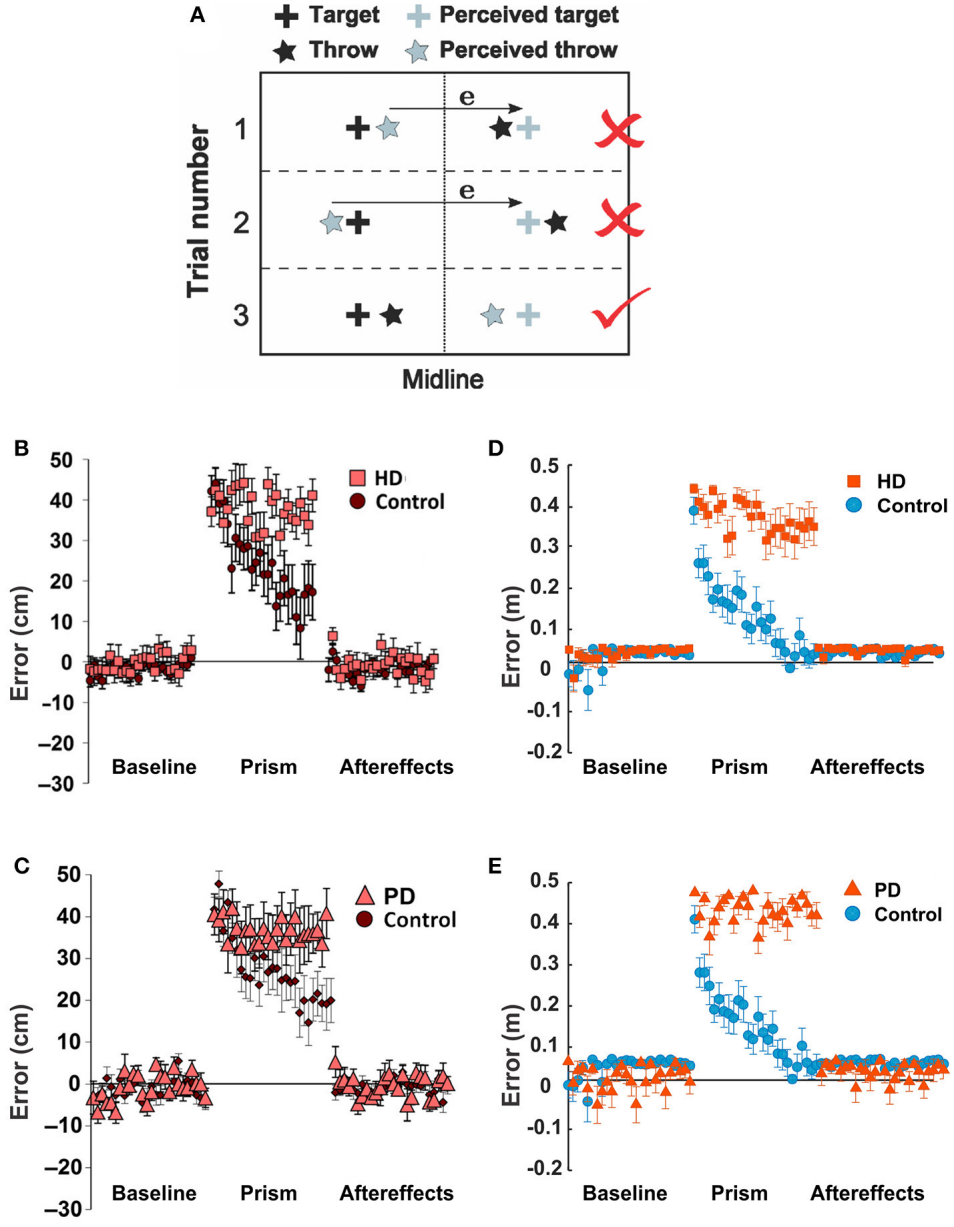
**Parkinson's and Huntington's effects on non-error based learning. (A–C)** Reproduced from Gutierrez-Garralda et al. (2013) with permission. **(A)** Throwing task. To hit the target (black cross) after the introduction of dove reversing prisms, participants had to throw 40 cm left of the perceived target (gray cross). Trial 1, 2: if participants used the error (e) they would end up throwing the ball further away from the target, even though their visual feedback would be nullified. Throwing to the same side of the target would be rewarded, but the error would be higher. Trial 3: the error should be ignored. **(B,C)** Non-error based motor adaptation shown as error distance vs. trial number in Huntington's Disease (HD) and Parkinson's Disease (PD) patients. **(D,E)** Same conditions simulated by the model. In the simulation, two sensory cues were used (C1 and C2); the cues are pre-associated with target actions T1 and T2, corresponding to the actual and perceived targets, respectively. To mimic the dove reversing prisms, during the perturbation reaching to T1 in response to C2 was rewarded. Thus, for the baseline period, only C1 was activated; for the perturbation period, only C2 was activated; and for the aftereffect period, only C1 was activated as in the baseline. **(D)** For the HD condition, the activity of D2-MSNs was suppressed by 90%. **(E)** For the PD condition, the learning rates for both D1 and D2 were reduced by 90%. See Methods for details.

From Figures 7B,C, we can see that both HD and PD patients showed deficits in learning during visual perturbation with the prism while the healthy subjects in the control group could gradually adapt to the perturbation in favor of reducing errors (Gutierrez-Garralda et al., 2013).

Similar to experimental observations, the simulations of HD and PD conditions demonstrated the model's inability to adapt to the perturbation and reduce errors in both cases (Figures 7D,E). The mechanisms, however, were different. As described in Methods, we simulated the PD conditions by a reduction in the learning rates for both D1 and D2 striatal populations mimicking a reduced dopaminergic signal. Therefore, the LTP in the D2- and/or D1-MSNs was not sufficient to suppress the previously habituated response and reinforce the more rewarding actions in 25 trials. Simulated HD conditions, however, assumed suppression of the D2 MSN activity, which implies inability of the model to overcome the previously acquired habit (see above). Therefore, the dysfunction of the indirect pathway, whether due to impaired neuroplasticity or neuronal activity, seems to be most critical in both conditions. Note that our simulation of HD and PD conditions did not eliminate initial acquisition and habituation during longer preliminary training period, as we used only partial (rather than complete) disruption of the described mechanisms.

#### Disruption of reward based learning by GPi blockade

**Figure 8:**
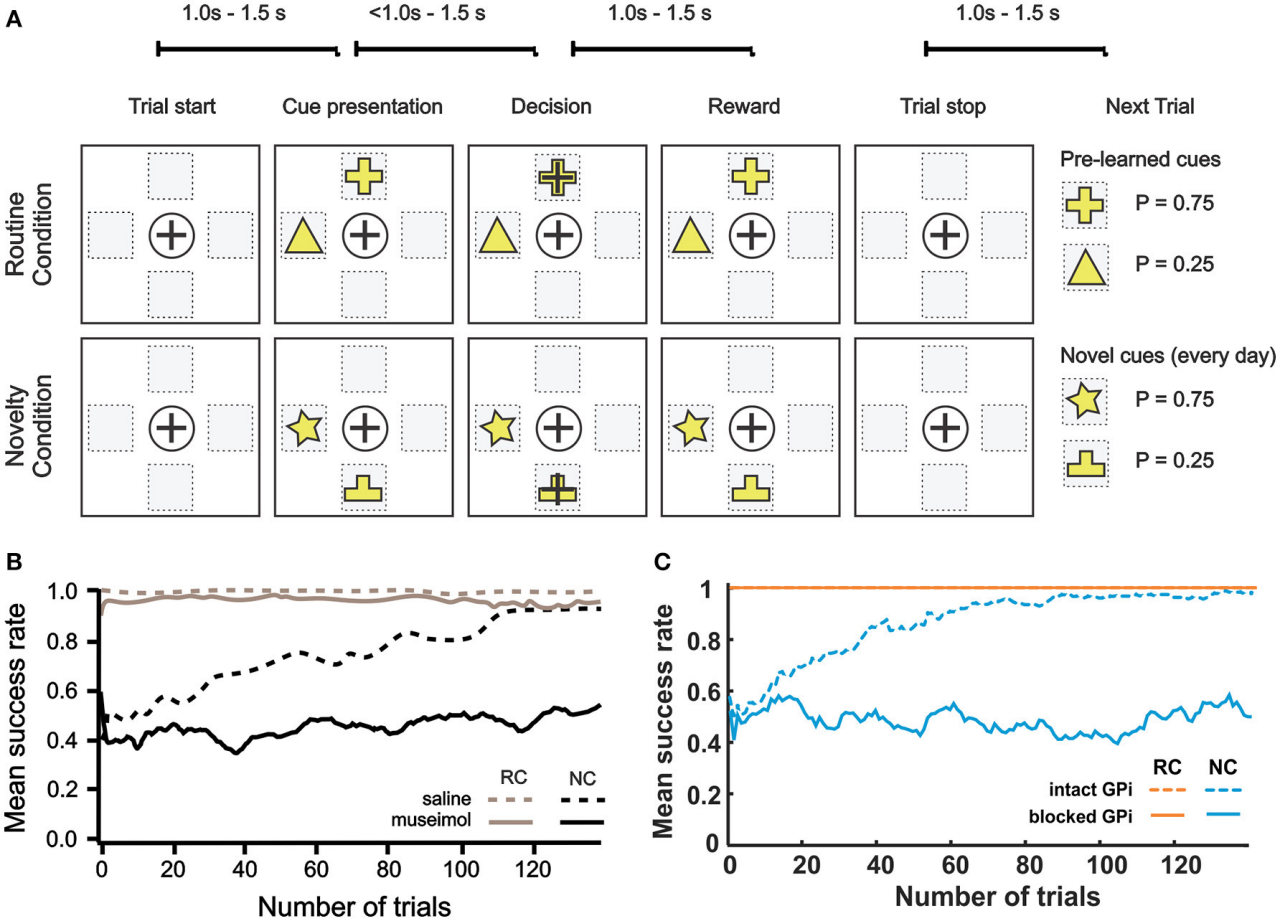
**Effect of GPi blockade. (A–B)** Reproduced from Piron et al. (2016) with permission. **(A)** Experiment protocol. One session = 250 trials = 25 blocks (1 block = 10 trials). The routine condition (RC) and novelty condition (NC) were alternated block by block during the session. In each trial, 2 buttons were displayed simultaneously in randomly chosen 2 of 4 selectable positions on the screen. The monkey showed its choice by moving the cursor to one of the buttons and was rewarded with a predefined probability that depended on the choice. The RC was pre-learned condition with fixed reward probabilities to achieve automatic/reflexive decision making. In the NC, two figures on randomly chosen buttons have the same reward probabilities (P_1_ = 0.75 and P_2_ = 0.25, respectively) as in the RC, but the symbols were different to assess learning capability and deliberative goal-oriented decision making. **(B)** Mean success rate for a monkey across trials. The curve is smoothed using a moving average filter of 10 consecutive trials. Monkeys' performances are almost optimal in the RC (gray), after saline (dashed line), or muscimol (solid line) injections. In the NC (black), the monkey is able to learn after saline injection (dashed black line), but not after the inactivation of GPi by muscimol injection (black solid line). **(C)** Corresponding model simulations. The mean success rate was calculated across 20 experiments with a moving average filter of 10 consecutive trials. To mimic muscimol injection, we zeroed GPi outputs.

Figure 8B shows experimental results from one of two monkeys from Piron et al. (2016) study (see Methods and the original publication for details). In the RC, with saline injection, the monkey performed optimally so that the success rate was almost 100%. Importantly, after muscimol injection, the success rate was close to 100% too, which implies that BG integrity is not required for habituated responses. In the NC, with saline injection, the success rate gradually increased from the initial 0.4 to 0.9 within 120 trials, which means that the monkey learned to favor the more rewarding label on the button in the NC. However, after muscimol injection, the monkey could not learn the NC so that the success rate stayed between 0.4 ~ 0.6 based on random selection of one of two highlighted buttons. In summary, monkeys in this experiment performed pre-learned decision making in the RC equally well before and after the GPi blockade but could not learn the novel associations after the GPi blockade.

Our simulated results are shown in Figure 8C. In the RC, both success rates were 1 regardless of whether the GPi was blocked or not. In the NC, the success rate stayed between 0.4 ~ 0.6 after the GPi blockade for all trials, and the success rate with intact GPi gradually increased from about 0.5 to 0.9 within 80 trials. In this simulation, the model behavior was qualitatively similar to the results described in Piron et al. experiment with a very close learning timescale.

## Discussion

This theoretical study is aimed at bridging the gap between the classical view of BG function and non-error based motor adaptation. To guide goal-directed movements, the motor control system in our body uses the internal model for an error-driven adjustment, and visuomotor learning usually involves this adjustment (Shadmehr et al., 2010). This type of supervised learning is attributed to the cerebellum (Izawa et al., 2012). However, under certain conditions the information about errors may be unavailable or misinterpreted (see Gutierrez-Garralda et al., 2013 and references therein). It has been shown that under such conditions motor adaptation in response to perturbation was still possible based on reinforcement learning mechanisms (Izawa and Shadmehr, 2011), which are believed to be mediated solely by the BG.

Behavioral experiments studying reinforcement learning mechanisms assume that a choice has to be made between several differentially rewarding behavioral options. Unlike decision-making tasks, motor learning does not imply a small or finite number of possible choices. The only constraint is the context of the task, e.g., reaching from a fixed initial position to an unknown destination, or pushing a joystick in an unknown direction. Moreover, even simple motor behaviors, like reaching movements, involve complex activation patterns of multiple muscles receiving inputs from many spinal motoneurons, whose activity is formed with input signals from the cortex and proprioceptive feedback signals from the muscles, interacting with a spinal network of interneurons.

Our objective was to create a scalable model with virtually unlimited number of possible actions. As the context, we chose center-out reaching movements performed in a horizontal plane. We used a previously published model of the neural control system that controls a two-joint arm through six muscles (Teka et al., in press). Unlike other similar models (Lillicrap and Scott, 2013; Mannella and Baldassarre, 2015), to calculate cortical activity corresponding to different movements, we explicitly solved an inverse problem based on the given arm dynamics. Accordingly, for every possible reaching movement we could calculate the corresponding motor program represented by the activity profiles of the neuronal pools in the motor cortex responsible for activation of different muscles.

### Mechanism of action selection

The classical view of action selection is that different motor actions are gated by thalamocortical relay neurons (see Frank, 2005 and references therein). In previous models, it was assumed that the action selection process results in activation of only one relay neuron corresponding to the selected action. In case of an arbitrarily large number of possible actions, this assumption does not seem plausible. In the presented model, we propose that relay neurons can be activated at different rates, and their firing rates define contributions of different motor programs to the resulting motor response. More specifically, in our model cortical input to the spinal network is implemented as a linear combination of all possible motor programs in the given context with coefficients defined by the firing rates of corresponding thalamocortical relay neurons. This linear combination can be viewed as an aggregate input to the spinal network from the cortical motoneurons exhibiting activity profiles corresponding to different motor behaviors, e.g., reaching movements in different directions. Interestingly, this implementation is consistent with previous experimental observations that during reaching movements in different directions the same motocortical neurons are activated to different extent, and the direction of movement correlates well with a population vector (Georgopoulos et al., 1986, 1988).

The classical concept of BG function suggests that the BG network is instrumental in performing a behavioral choice that maximizes reward. This action selection process results in activation of thalamic relay neurons corresponding to the selected action and suppression of neurons gating other behaviors. Per this concept, each action is dedicated to specific neurons in different BG nuclei. Their focused interconnections form action-related loops which start at the cortex, bifurcate in the striatum into direct and indirect pathways converging on the GPi, and feed back to the cortex through the thalamus. Action preference is facilitated by increased excitatory projections from sensory cortical neurons representing the stimulus to direct pathway striatal neurons (D1 MSNs). Suppression of unwanted competing actions is assumed to occur because of lateral inhibition among the loops at some level of the network in a winner-takes-all manner.

The classical model predicts that novel cue-action associations acquired based on reinforcement learning rely on BG network integrity. However, multiple experimental studies have shown that pharmacological blockade of GPi, the BG output structure, does not lead to significant impairments in performing well-learned tasks (Desmurget and Turner, 2010; Piron et al., 2016). Consistent with these experiments, it was suggested that acquired associations may become “direct” projections within the cortex bypassing the BG network (Ashby et al., 2010). Unfortunately, this does not explain how suppression of competing actions is performed after the BG output is blocked.

In our implementation of the model, competing actions are suppressed by lateral inhibition in the population representing thalamocortical neurons, independent of BG network integrity. Our model does not elaborate on mechanisms of this inhibition. However, one can speculate that GABAergic neurons in the thalamic reticular nucleus may serve their purpose by receiving focused excitatory inputs from thalamocortical neurons and sending diffused inhibition back to them (Pinault and Deschênes, 1998) or fast-spiking, local GABAergic inhibitory interneurons in the neocortex may play the role since they are directly receive thalamocortical afferents, contact excitatory cortical neurons in a disynaptic way and exert fast and strong inhibition (Miller et al., 2001; Hull and Scanziani, 2007).

### Mechanisms of cue-action association and habituation

In the model, novel cue-action associations are formed based on reinforcement learning in the striatum. Eventually, the preferable behavior is reliably selected due to potentiated projections from PFC neurons, corresponding to the provided stimulus, to D1 MSNs, corresponding to the preferred behavior. On a longer timescale, repetitive execution of the same action in response to the same stimulus leads to habituation of the response via LTP of the direct projections between the corresponding PFC and PMC neurons based on Hebbian learning. At the same time, due to degradation of synaptic connections between the PFC and striatum in absence of reinforcement, BG involvement in the action selection process gradually decreases. Eventually, the behavior becomes a habit, which is automatically selected solely based on direct cortico-cortical projections.

After the learned cue-action is completely habituated, i.e., transferred from the BG to the cortical network, the integrity of the BG and/or their output nuclei becomes less critical for action selection, because neither action selection nor action discrimination processes are performed by these structures. This explains related experimental findings (Desmurget and Turner, 2010). However, suppression of BG output should prevent acquisition of novel associations, which is also found in experimental evidence (Piron et al., 2016).

### The role of the indirect pathway

Our model predicts that proper functioning of the indirect pathway is critically important for reversal learning. If a habituated behavior is no longer appropriate, plasticity in the indirect pathway provides a mechanism to suppress the habit. In this case, negative reinforcement delivered as a reduction in dopamine tone leads to LTP of cortical projections to the D2 MSNs in the striatum. Next time the same stimulus is presented, increased excitatory input to D2 MSNs results in their stronger activation, which prevents corresponding thalamocortical neurons from activation even though they receive a “direct” excitatory input from the PFC. This prohibits execution of the habit and permits the system to explore new potentially beneficial behavioral responses. Accordingly, the model predicts that any malfunctioning of the indirect pathway neurons, e.g., altered activity of the nodes and/or impaired neurotransmission, would result in impaired reversal learning but not in the acquisition of completely novel cue-action associations. In the model, the indirect pathway is mediated by D2 MSNs, GPe and STN (Figure 3). At least two of these structures are found to be affected by Huntington's Disease which at early stages leads to neurodegeneration of D2 MSNs (Reiner et al., 2011) and to reduced excitability of the STN neurons in two different mouse models of the disease (Callahan and Abercrombie, 2015a,b). We simulated both conditions and showed similarities between the model's behavior and the outcomes of experiments by Gutierrez-Garralda et al. (2013), where they demonstrated impairment of reversal learning in HD patients.

Mandali et al. (2015) exploited the idea that the STN-GPe loop, a coupled excitatory-inhibitory network in the indirect pathway, may provide a basis for the exploration mechanism. Via simulations, they suggested that the decrease in exploration is driven by a change in synchrony levels in the STN-GPe circuit, which could be a possible explanation for a reduced exploratory activity in PD patients. This may represent another interesting aspect of the indirect pathway function, in addition to those described in our study.

## Conclusion

In this study, we described a model of the interaction between the BG network and the motor control system, which provides a mechanistic interpretation to non-error based motor adaptation phenomena. We propose that the context of the motor task is represented in the cortex as a reservoir of all possible movements, and the BG synthesize rather than select the response behavior by modulating the activity of thalamocortical relay neurons based on reinforcement learning mechanisms. To explain the experiments with pharmacological suppression or lesions of BG output nuclei, we suggest that lateral inhibition, responsible for action discrimination, occurs in the thalamocortical network. Finally, we predict that any impairments in the indirect pathway function would lead to difficulties with suppression of well-learned responses in case they become inappropriate, which may be studied using reversal learning experimental paradigms.

## Author contributions

TK, KH, DT contributed equally. Conceptualization: SM, IR, YM. Methodology: TK, KH, DT, SM, YM. Software: TK, KH, DT, SM, YM. Validation: TK, KH, DT, SM, WB, RC, EL, YM. Formal analysis: TK, KH, DT, SM, YM. Investigation: TK, KH, DT, SM, YM. Resources: SM, IR, YM. Data curation: TK, KH, DT, WB, RC, EL, SM. Writing (original draft preparation): TK, KH, YM. Writing (review and editing): TK, KH, DT, WB, RC, EL, SM, IR, YM. Visualization: TK, KH, SM. Supervision: IR, YM. Project administration: IR, YM. Funding acquisition: IR, YM.

## Funding

This work is supported by CHDI Foundation #A-8427.

### Conflict of interest statement

The authors declare that the research was conducted in the absence of any commercial or financial relationships that could be construed as a potential conflict of interest.
